# Two Quantum Proxy Blind Signature Schemes Based on Controlled Quantum Teleportation

**DOI:** 10.3390/e24101421

**Published:** 2022-10-05

**Authors:** Qiming Luo, Tinggui Zhang, Xiaofen Huang, Naihuan Jing

**Affiliations:** 1 School of Mathematics and Statistics, Hainan Normal University, Haikou 571158, China; 2 Key Laboratory of Data Science and Smart Education, Ministry of Education, Hainan Normal University, Haikou 571158, China; 3 Department of Mathematics, North Carolina State University, Raleigh, NC 27695, USA; 4 College of Sciences, Shanghai University, Shanghai 200444, China

**Keywords:** quantum teleportation, quantum signature, proxy signature, blind signature, 03.67.-a, 02.20.Hj, 03.65.-w

## Abstract

We present a scheme for teleporting an unknown, two-particle entangled state with a message from a sender (Alice) to a receiver (Bob) via a six-particle entangled channel. We also present another scheme for teleporting an unknown one-particle entangled state with a message transmitted in a two-way form between the same sender and receiver via a five-qubit cluster state. One-way hash functions, Bell-state measurements, and unitary operations are adopted in these two schemes. Our schemes use the physical characteristics of quantum mechanics to implement delegation, signature, and verification processes. Moreover, a quantum key distribution protocol and a one-time pad are adopted in these schemes.

## 1. Introduction

Following the in-depth development of quantum key distribution (QKD) [1,2,3], various quantum cryptographic protocols have been developed, such as the quantum secure direct communication [4,5,6,7], quantum private query [8,9,10], quantum secret sharing [11,12,13], quantum multiparty secure computations [14,15], quantum authentication [16,17,18], quantum signature [19], and others. To ensure the safety of quantum cryptographic protocols, some attacking strategies on quantum cryptography protocols have been proposed, such as intercepted-resend [20,21], entanglement-swapping [22,23], teleportation [24], dense-coding [25,26,27], channel-loss [28], denial-of-service [29,30], correlation-extractability [31,32], trojan-horse [33,34], information-leakage [35], collusive attacks [36], and others. These effective attack strategies helped the design of new quantum cryptography schemes that are more secured.

In recent years, many different quantum signature schemes have been proposed for different application environments, including (but not limited to) quantum blind [37,38], quantum proxy [39,40], quantum multiple [41,42], quantum group [43,44], and others. In 2013, Zhang et al. proposed a secure quantum group signature scheme based on the Bell state [45], and they also presented the cryptanalysis of the quantum group signature protocol [46]. These signature schemes can be applied to e-commerce or other related fields.

The digital signature, which was independently introduced by Diffe [47] and Merkle [48], has been an important branch of cryptography. Digital signatures constitute a very important research topic in classic cryptography as it has many applications in real life. The security of classical signature schemes is based on complex mathematical problems, such as factorization and discrete logarithm problems. However, classic signatures are becoming increasingly vulnerable as quantum algorithms develop further. Fortunately, in quantum information processing and computation, quantum cryptography can provide secure communication based on quantum mechanics, especially when based on the no-cloning theorem. Inspired by these properties, some progress has been achieved in the field of quantum signatures [49,50,51].

Proxy signatures, which allow the original signer to authorize the proxy signer to sign messages on his behalf, plays a key role in cryptography. Since Mambo et al. [52] proposed the concept of the proxy signature in 1996, some signature schemes have been proposed. Wang et al. [53] proposed a one-time proxy signature scheme without decoherence. Yang et al. [54] subsequently indicated that this scheme could not meet the security requirements of non-forgery and nonrepudiation. However, Wang et al. [55] showed in 2015 that this conclusion was not appropriate. Recently, Cao et al. [39,56] presented two quantum proxy signature schemes based on some genuine six- and five-qubit entangled states. However, Zhang et al. [57] suggested that receivers could forge valid signatures. In 2015, Tian et al. [58] presented a quantum multi-proxy blind signature scheme based on four-qubit entangled states with fewer resources.

The blind signature is a special digital signature that can protect the anonymity of the message owner to ensure privacy. In blind signatures, the message owner can always obtain the real signature of his message, even if the signer knows nothing about the content of his signature. Blind signatures can be classified into weak and strong blind signatures based on whether the signer can track the message owner. Chaum proposed the first blind signature scheme in 1983 based on the complexity of factoring large integers [59]. However, it was easily broken with the emergence of quantum computers. In 2010, Su et al. [38] presented a weak blind quantum signature scheme based on a two-state vector formalism. They used EPR pairs to implement the signature process, and applied the characteristics of the two-state vector form to implement the verification scheme. In recent years, quantum signature schemes have received extensive attention.

In this study, we propose two quantum proxy blind signature schemes based on controlled quantum teleportation. The first scheme takes advantage of the correlation of the maximal EPR and four-qubit entangled states, Bell-state measurements (BMs), {|0〉,|1〉} basis measurements, Hadamard operation, quantum-key distribution, and unitary operations. In this scheme, two particles are delivered to the original signer, Alice. The proxy signers Charlie and David hold one particle each. The remaining two particles are distributed to the verifier, Bob. The second scheme is bidirectional, taking advantage of the correlation of the five-qubit cluster state, BMs, {|+〉,|−〉} basis measurements, quantum-key distribution, and unitary operations. In this scheme, two particles are delivered to the original signer and verifier, Alice. The proxy signer Charlie holds one particle. The other two particles are distributed to the verifier and original signer, Bob. We used the quantum key distribution and a one-time pad to guarantee the unconditional security and signature anonymity. It is shown to be unconditionally secured, i.e., may not be forged or modified in any way by the receiver or attacker. In addition, it may neither be disavowed by the signatory, nor denied by the receiver.

## 2. Controlled Quantum Teleportation of the First Scheme

Our multiproxy blind signature scheme is based on the controlled quantum teleportation that uses the maximal EPR state and maximal four-qubit entangled states as its quantum channel. It is given by

(1) $$\begin{array}{cc} {|\psi \rangle }_{3456} & =\frac{1}{2\sqrt{2}}(|0000\rangle -|0111\rangle +|1001\rangle -|1110\rangle \\ & +|0110\rangle +|0001\rangle +|1111\rangle +{\left|1000\rangle \right)}_{3456}, \end{array}$$

(2) $$\begin{array}{cc} {|\psi \rangle }_{78} & =\frac{1}{\sqrt{2}}(|00\rangle +{\left|11\rangle \right)}_{78}. \end{array}$$

Suppose that the quantum state of particle carrying message in Alice is

(3) $$\begin{array}{c} {|\phi \rangle }_{12}=(\alpha |00\rangle +\beta |01\rangle +\gamma |10\rangle +{\delta |11\rangle )}_{12}, \end{array}$$

where the unknown coefficients α, β, γ and δ satisfy ${\left|\alpha \right|}^{2}+{\left|\beta \right|}^{2}+{\left|\gamma \right|}^{2}+{\left|\delta \right|}^{2}=1$.

This controlled quantum teleportation scheme involves the following four partners: the sender (Alice), two controllers (Charlie and David), and the receiver (Bob). Alice holds particles (1,2,3,7), Bob holds particles (6,8), and Charlie and David own particles 4 and 5. Therefore, the quantum state |Ψ〉 of the entire system composed of particles (1,2,3,4,5,6,7,8) is as follows

(4) $$\begin{array}{c} |\Psi \rangle ={|\phi \rangle }_{12}⊗{|\psi \rangle }_{3456}⊗{|\psi \rangle }_{78}. \end{array}$$

The steps of the controlled quantum teleportation are as follows (the detailed process is described in reference [60]).

(1) Alice performs BMs on particles (1,3) and (2,7). These are 16 possible outcomes.
(2) Alice sends her measurement outcomes to Bob through a secure quantum channel. Bob then performs a corresponding unitary operation on particles (6,8).
(3) If Charlie and David agree with Alice and Bob to complete their teleportation process, they perform the Hadamard operation on their particles, respectively. Subsequently, they perform a {|0〉,|1〉} based measurement on their particles. They then send their measurement outcomes to Bob through a secure quantum channel.
(4) When the two measurements are the same, Bob will obtain the quantum state transferred from Alice. When the two measurements are different, Bob just needs to apply the unitary operation $\sigma_{x}$ on particle 6, whereupon the same receiver obtains the quantum state transferred from Alice.

## 3. Quantum Multiproxy Blind Signature Scheme

In our scheme, the participants are defined as follows.

(i) Alice: the original signer; (ii) Bob: the message receiver; (iii) Charlie and David: two proxy signer; and (iiii) Trent: the message verifier and trusted party.

The detailed procedure of our scheme can be described as follows.

### 3.1. Initial Phase

(i) QKD. Alice shares the secret key $K_{AB}$ with Bob. In addition, Bob establishes the secret keys $K_{BC}$ with Charlie and $K_{BD}$ with David. Moreover, Trent shares the secret keys $K_{TA}$ with Alice, $K_{TB}$ with Bob, $K_{TC}$ with Charlie, and $K_{TD}$ with David. These distribution tasks can be fulfilled via QKD protocols, which have been proved to be unconditionally safe.
(ii) Quantum Channel Establishment. Bob produces t+l quantum states ${|\psi \rangle }_{3456}$ and ${|\psi \rangle }_{78}$. He sends particles (3,7) to Alice, particle 4 to Charlie, and particle 5 to David, leaving particles (6,8) to himself.
(iii) To ensure security of the quantum channel, Bob arranges eavesdropping checks.

### 3.2. Blinding the Message Phase

(i) Alice converts her message *m* into an N-bit sequence and records m={m(1),m(2),…,m(j),…,m(N)}. Subsequently, Alice sends the binary sequence *m* to Trent.
(ii) Alice transforms *m* to H(m) by using a Hash function, where *H*: ${\left\{0,1\right\}}^{N}\to {\left\{0,1\right\}}^{n}\left(N\gg n\right)$, and Trent also knows the Hash function *H*. She then blinds the message based on $M\left(i\right)=K_{TA}⊕m\left(i\right),i=1,2,\ldots ,n$, where ⊕ is the XOR operation. She can identify an appropriate Hash function, such that *n* is an even umber. Let $l=\frac{n}{2}$.
(iii) Alice produces $\frac{n}{2}$ quantum states ${|\phi \rangle }_{12}=(\alpha |00\rangle +\beta |01\rangle +\gamma |10\rangle +{\delta |11\rangle )}_{12}$, denoted as ${|\phi \rangle }_{12}\left(1\right)$, ${|\phi \rangle }_{12}\left(2\right)$, …, ${|\phi \rangle }_{12}\left(\frac{n}{2}\right)$. If $m\left(i\right)={00,|\phi \rangle }_{12}=|00\rangle$; If $m\left(i\right)={01,|\phi \rangle }_{12}=|01\rangle$; if $m\left(i\right)={10,|\phi \rangle }_{12}=|10\rangle$; if $m\left(i\right)={11,|\phi \rangle }_{12}=|11\rangle$.

### 3.3. Authorizing and Signing Phases

In our scheme, we used a one-time pad as the encryption algorithm to ensure unconditional security.

(i) If Alice agrees with Charlie and David as her proxy signers to sign the message, she will help to perform controlled teleportation. Alice performs the BMs on particles (1,3) and (2,7) and records the measurement outcomes as $S_{A}$. She then encrypts $S_{A}$ with the key $K_{AB}$ to obtain the secret message $E_{K_{AB}}\left\{S_{A}\right\}$. Alice sends the message $E_{K_{TA}}\left\{S_{A}\right\}$ to Trent via the quantum channel. Similarly, Alice sends the message $E_{K_{AB}}\left\{S_{A}\right\}$ to Bob via the quantum channel.
(ii) After Bob has received the message $E_{K_{AB}}\left\{S_{A}\right\}$, he decrypts it with his $K_{AB}$ to obtain the message $S_{A}$. Bob then performs corresponding unitary operations on particles (6,8). After this, he encrypts $S_{A}$ with the keys $K_{BC}$ and $K_{BD}$ to obtain the secret messages $E_{K_{BC}}\left\{S_{A}\right\}$ and $E_{K_{BD}}\left\{S_{A}\right\}$, respectively. Bob sends the messages $E_{K_{BC}}\left\{S_{A}\right\}$ and $E_{K_{BD}}\left\{S_{A}\right\}$ to Charlie and David, respectively, via the quantum channel.
(iii) After Charlie and David have received the messages $E_{K_{BC}}\left\{S_{A}\right\}$ and $E_{K_{BD}}\left\{S_{A}\right\}$, they decrypt it with their keys $K_{BC}$ and $K_{BD}$ to obtain the message $S_{A}$. They then perform the Hadamard operation on their particles. Subsequently, they perform a {|0〉,|1〉} based measurement on their particles, and they note the measurement outcomes as $S_{C}=\left\{c\left(1\right),c\left(2\right),\ldots c\left(i\right),\ldots ,c\left(\frac{n}{2}\right)\right\}$ and $S_{D}=\left\{d\left(1\right),d\left(2\right),\ldots d\left(i\right),\ldots ,d\left(\frac{n}{2}\right)\right\}$. Charlie then encrypts {$S_{A}$, $S_{C}$} with the use of the key $K_{BC}$ to obtain the secret message $S_{1}=E_{K_{BC}}\left\{S_{A},S_{C}\right\}$, and David also encrypts {$S_{A}$, $S_{D}$} with the key $K_{BD}$ to obtain the secret message $S_{2}=E_{K_{BD}}\left\{S_{A},S_{D}\right\}$. They send the messages $S_{1}$ and $S_{2}$ to Bob as the proxy authorization. Similarly, they send {$S_{A}$, $S_{C}$} and {$S_{A}$, $S_{D}$} to Trent by $K_{TC}$ and $K_{TD}$.

### 3.4. Verifying Phases

(i) Bob receives the messages $S_{A}$, $S_{C}$ and $S_{D}$ by using the keys $K_{BC}$ and $K_{BD}$ to decrypt $S_{1}$ and $S_{2}$. If $S_{A}$ in the proxy signature does not match that sent by Alice, the signature verification process will be terminated, and the signature will be declared invalid. Otherwise, the process continues based on the following steps.
(ii) According to $S_{C}$ and $S_{D}$, Bob performs an appropriate unitary operation on particle 6 to replicate the unknown state ${|\phi \rangle }_{12}$ which carries the messages. He then sends the state ${|\phi \rangle }_{12}$ to Trent.
(iii) Trent encodes the quantum state ${|\phi \rangle }_{1^{\prime }2^{\prime }}$ to obtain the message $M^{\prime }$. If $M^{\prime }=M$, he confirms a series of signatures and messages $\left(m,S_{C},S_{D}\right)$. Otherwise, Trent rejects it.

## 4. Security Analysis and Discussion of the First Scheme

In this section, we will demonstrate that our scheme meets the following security requirements in accordance with reference [57].

### 4.1. Message Blindness

Alice blinded the message *m* to *M*. Proxy signers Charlie and David could not obtain the contents of the messages because Alice used hash functions and XOR operations to blind them. Therefore, Charlie and David could not know the content of the information she signed.

### 4.2. Impossibility of Denial

In this scheme, we prove that Alice cannot refuse her delegation, and Charlie and David cannot refuse their signatures. If the signature is verified, the signer cannot reject its signature or message for the following reasons: (1) the signature is encrypted by the key, and (2) the entangled states have stable coherence among particles. Only by measuring their own particles can they achieve teleportation. All keys are distributed based on the QKD protocol, which has been unconditionally proved. All messages are sent through secure quantum channels. Therefore, Alice cannot refuse her delegation, nor can Charlie and David refuse their signatures.

### 4.3. Impossibility of Forgery

Firstly, we assume that Trent is completely credible. We can prove that Charlie and David cannot forge Alice’s signature. In our scheme, trusted Trent can prevent these attacks. Suppose Charlie tries to forge Alice’s signature. Even if he knows the key, he cannot forge any signature because Charlie’s forgery attack will be detected by Trent. Similarly, David could not input Alice’s signature for Charlie’s signature. In other words, internal attackers cannot forge their signatures.

Secondly, we assumed that there was an external attacker (Eve). Eve cannot obtain the secret key. Therefore, she cannot forge the signatures of Alice, Charlie, and David. We assumed that Eve could obtain the secret key randomly and generate a valid signature with the probability $\frac{1}{2^{n}}$ tending to zero when *n* tends to infinity. At the same time, we realized the eavesdropping detection function in the construction stage of the quantum channel. Therefore, the signing process will only continue when the channel is secure; otherwise, the signing process will terminate. Therefore, Eve cannot forge their signatures.

## 5. Controlled Quantum Teleportation of the Second Scheme

Our two-way proxy blind signature scheme is based on controlled quantum teleportation that uses the five-qubit cluster state as its quantum channel. It is given by,

(5) $$\begin{array}{c} {|\phi \rangle }_{12345}=\frac{1}{2}(|00000\rangle +|00111\rangle +|11101\rangle +|11010\rangle ). \end{array}$$

Suppose that the quantum state of the particle-carrying message for Alice is

(6) $$\begin{array}{ccc} {|\phi \rangle }_{A} & = & (\alpha |0\rangle +{\beta |1\rangle )}_{A},\\ {|\phi \rangle }_{B} & = & (\gamma |0\rangle +{\delta |1\rangle )}_{B}, \end{array}$$

where the unknown coefficients α, β, γ and δ satisfy ${\left|\alpha \right|}^{2}+{\left|\beta \right|}^{2}=1$ and ${\left|\gamma \right|}^{2}+{\left|\delta \right|}^{2}=1$.

This controlled quantum teleportation involves the following three partners: the sender and receiver Alice, sender and receiver Bob, and the controller Charlie. Alice holds particles (1,5,A), Bob holds particles (2,3,B), and Charlie owns particle 4. Thus, the quantum states |Ψ〉 of the entire system takes the following form

(7) $$\begin{array}{c} |\Psi \rangle ={|\phi \rangle }_{B}⊗{|\phi \rangle }_{A}⊗{|\phi \rangle }_{12345}. \end{array}$$

The steps of the controlled quantum teleportation are as follows (the detailed process is described in reference [61]).

(1) Alice performs BMs on particles (A,1) and Bob performs BMs on particles (B,3), respectively. These are 16 types of possible outcomes.
(2) Alice sends her measurement outcomes to Bob and Charlie through the secured quantum channel. Bob sends his measurement outcomes to Alice and Charlie.
(3) If Charlie agrees with Alice and Bob to complete their two-way teleportation processes, he performs a {|+〉,|−〉} based measurement on particle 4. He then sends his measurement outcomes to Alice and Bob through the secured quantum channel.
(4) Bob performs a corresponding unitary operation on particle 2, whereupon Bob receives the quantum state transferred from Alice. The same applies for Alice who receives the quantum state for particle 5 transferred from Bob.

## 6. Quantum Two-Way Proxy Blind Signature Scheme

In our scheme, participants are defined as follows:

(i) Alice and Bob: the original signers; (ii) Charlie: the proxy signer; and (iii) Trent: the message verifier and the trusted party.

The detailed procedure of our scheme is as follows.

### 6.1. Initial Phase

(i) QKD. Alice shares the secret key $K_{AB}$ with Bob and shares the secret key $K_{AC}$ with Charlie. In addition, Bob establishes the secret key $K_{BC}$ with Charlie. Moreover, Trent shares the secret keys $K_{TA}$ with Alice, $K_{TB}$ with Bob, and $K_{TC}$ with Charlie. These distribution tasks can be fulfilled via QKD protocols, which have been proven to be unconditionally secured.
(ii) Quantum Channel Establishment. Bob produces p+q quantum states ${|\phi \rangle }_{12345}$. He sends particles (1,5) to Alice, particle 4 to Charlie, and leaves particles (2,3) to himself.
(iii) To ensure the security of the quantum channel, Bob arranges eavesdropping checks.

### 6.2. Blinding the Message Phase

(i) Alice converts the message $m^{\prime }$ into a K-bit sequence and records $m^{\prime }=\{m^{\prime }\left(1\right),m^{\prime }\left(2\right),\ldots ,$$m^{\prime }\left(j\right),\ldots ,m^{\prime }\left(K\right)\}$. Subsequently, Alice sends the binary sequence $m^{\prime }$ to Trent. Similarly, Bob converts the message $m^{\prime \prime }$ into an R-bit sequence and records $m^{\prime \prime }=\{m^{\prime \prime }\left(1\right),m^{\prime \prime }\left(2\right),$$\ldots ,m^{\prime \prime }\left(j\right),\ldots ,m^{\prime \prime }\left(R\right)\}$. Subsequently, Bob sends the binary sequence $m^{\prime \prime }$ to Trent.
(ii) Alice converts $m^{\prime }$ to $H^{\prime }\left(m^{\prime }\right)$ by using the Hash function, where $H^{\prime }$, ${\left\{0,1\right\}}^{K}\to {\left\{0,1\right\}}^{q}\left(K\gg q\right)$, and Trent also knows the Hash function $H^{\prime }$. She then blinds the message by $M^{\prime }\left(i\right)=K_{TA}⊕m^{\prime }\left(i\right),i=1,2,\ldots ,q$, where ⊕ is the XOR operation. Similarly, Bob converts $m^{\prime \prime }$ to $H^{\prime }\left(m^{\prime \prime }\right)$ by using the Hash function, where $H^{\prime }$: ${\left\{0,1\right\}}^{K}\to {\left\{0,1\right\}}^{q}\left(K\gg q\right)$. He then blinds the message by $M^{\prime \prime }\left(i\right)=K_{TB}⊕m^{\prime \prime }\left(i\right),i=1,2,\ldots ,q$.
(iii) Alice produces *q* quantum states ${|\phi \rangle }_{A}=(\alpha |0\rangle +{\beta |1\rangle )}_{A}$, denoted as ${|\phi \rangle }_{A}\left(1\right)$,${|\phi \rangle }_{A}\left(2\right)$, …, ${|\phi \rangle }_{A}\left(q\right)$. Similarly, Bob produces *q* quantum states ${|\phi \rangle }_{B}=(\gamma |0\rangle +{\delta |1\rangle )}_{B}$, denoted as ${|\phi \rangle }_{B}\left(1\right)$,${|\phi \rangle }_{B}\left(2\right)$,…,${|\phi \rangle }_{B}\left(q\right)$.

### 6.3. Authorizing and Signing Phase

In our scheme, we used the one-time pad as the encryption algorithm to ensure unconditional security.

(i) If Alice agrees that Charlie can act as her proxy signer to sign the message, she will help the execution of controlled teleportation. Alice performs the BM on particles (A,1) and records the measured outcomes as $S_{A}$. She then encrypts $S_{A}$ with the keys $K_{AB}$ and $K_{AC}$ to obtain the secret messages $E_{K_{AB}}\left\{S_{A}\right\}$ and $E_{K_{AC}}\left\{S_{A}\right\}$. Alice sends the messages $E_{K_{AB}}\left\{S_{A}\right\}$ to Bob and $E_{K_{AC}}\left\{S_{A}\right\}$ to Charlie via the quantum channel. Similarly, If Bob agrees that Charlie can act as his proxy signer to sign the message, he will help the execution of controlled teleportation. Bob performs the BM on particles (B,3) and records the measuring outcomes as $S_{B}$. He then encrypts $S_{B}$ with the keys $K_{AB}$ and $K_{BC}$ to obtain the secret messages $E_{K_{AB}}\left\{S_{B}\right\}$ and $E_{K_{BC}}\left\{S_{B}\right\}$. Bob sends the messages $E_{K_{AB}}\left\{S_{B}\right\}$ to Alice and $E_{K_{BC}}\left\{S_{B}\right\}$ to Charlie via the quantum channel.
(ii) After Charlie has received the messages $E_{K_{AC}}\left\{S_{A}\right\}$ and $E_{K_{BC}}\left\{S_{B}\right\}$, he decrypts them with their keys $K_{AC}$ and $K_{BC}$ to obtain the messages $S_{A}$ and $S_{B}$. Charlie then performs a {|+〉,|−〉} based measurement on particle 4, and he notes the measured outcome as $S_{C}=\left\{c\left(1\right),c\left(2\right),\ldots c\left(i\right),\ldots ,c\left(q\right)\right\}$. Charlie encrypts {$S_{A}$, $S_{C}$} with the use of the key $K_{BC}$ to obtain the secret message $S_{1}=E_{K_{BC}}\left\{S_{A},S_{C}\right\}$, and also encrypts {$S_{B}$, $S_{C}$} with the use of the key $K_{BC}$ to obtain the secret message $S_{2}=E_{K_{BC}}\left\{S_{B},S_{C}\right\}$. He then sends the messages $S_{1}$ to Bob and $S_{2}$ to Alice as the proxy authorization. Similarly, Charlie sends {$S_{A}$, $S_{C}$} and {$S_{B}$, $S_{C}$} to Trent by using $K_{TA}$ and $K_{TB}$, respectively.

### 6.4. Verifying Phases

(i) Bob receives the messages $S_{A}$, $S_{C}$ by using the key $K_{BC}$ to decrypt $S_{1}$. If $S_{A}$ in the proxy signature does not match that sent by Alice, the signature verification process will be terminated, and the signature will be declared invalid. Otherwise, continue with the steps which follow (ii and iii). Similarly, Alice receives the messages $S_{B}$, $S_{C}$ by using the key $K_{AC}$ to decrypt $S_{2}$. If $S_{B}$ in the proxy signature does not match that sent by Bob, the signature verification process will be terminated, and the signature will be declared invalid. Otherwise, continue in accordance with the following steps.
(ii) According to $S_{A}$ and $S_{C}$, Bob performs an appropriate unitary operation on particle 2 to replicate the unknown state ${|\phi \rangle }_{A^{\prime }}$ which carries messages. Similarly, according to $S_{B}$ and $S_{C}$, Alice performs an appropriate unitary operation on particle 5 to replicate the unknown state ${|\phi \rangle }_{B^{\prime }}$ which carries messages. Alice and Bob then sends the states ${|\phi \rangle }_{B^{\prime }}$ and ${|\phi \rangle }_{A^{\prime }}$ to Trent.
(iii) Trent encodes the quantum state ${|\phi \rangle }_{A^{\prime }}$ and obtains the message $M^{\prime \prime \prime }$. He then compares it with $M^{\prime }$. If $M^{\prime \prime \prime }=M^{\prime }$, he confirms a series of signatures and messages $\left(m,S_{A},S_{C}\right)$. Otherwise, Trent rejects it. Similarly, Trent encodes the quantum state ${|\phi \rangle }_{B^{\prime }}$, obtains the message $M^{⁗}$ and compares it with $M^{\prime \prime }$. If $M^{⁗}=M^{\prime \prime }$, he confirms a series of signatures and messages $\left(m,S_{B},S_{C}\right)$. Otherwise, Trent rejects it.

## 7. Security Analysis and Discussion of the Second Scheme

In this section, we will demonstrate that our scheme meets the following security requirements.

### 7.1. Message Blindness

Alice blinded the message $m^{\prime }$ to $M^{\prime }$, and Bob blinded the message $m^{\prime \prime }$ to $M^{\prime \prime }$. Proxy signer Charlie was unable to obtain the contents of the message because Alice and Bob used hash functions and XOR operations to blind messages. Therefore, Charlie cannot know the contents of the information he signed.

### 7.2. Impossibility of Denial

We verified that Alice and Bob could not refuse their delegations, nor could Charlie refuse his signature. If the signature is verified, the signer cannot reject its signature or message. This is due to the following factors: (1) the signature is encrypted by the key, and (2) the entangled states have stable coherence among particles. Teleportation can be achieved only by measuring their own particles. All keys are distributed through the QKD protocol, which has been unconditionally proven. All messages are sent through secure quantum channels. Therefore, Alice and Bob cannot deny their authorization, and Charlie cannot refuse his signature.

### 7.3. Impossibility of Forgery

Firstly, we can prove that Charlie cannot forge Alice’s or Bob’s signatures because trusted Trent can prevent these attacks in our scheme. Suppose that Charlie tries to forge Alice’s signature. Even if he knows the key, he cannot forge any signature because Charlie’s forgery attack will be detected by Trent. Likewise, Charlie cannot forge Bob’s signature. In other words, internal attackers cannot forge signatures.

Secondly, we assumed that there was an external attacker (Eve). Eve cannot obtain the secret key. Therefore, she cannot forge their signatures. We also assumed that Eve could obtain the secret key randomly and generate a valid signature with the probability $\frac{1}{2^{n}}$ tending to zero when *n* tends to infinity. At the same time, we realized the eavesdropping detection function in the construction stage of the quantum channel. Therefore, the signing process will only continue when the channel is secure; otherwise, the signing will terminate. Therefore, Eve cannot forge their signatures.

## 8. Conclusions

In this study, we proposed two quantum proxy blind signature schemes based on controlled quantum teleportation. We presented a scheme for teleporting an unknown two-particle entangled state with a message from a sender (Alice) to a receiver (Bob) via a six-particle entangled channel. Additionally, we presented a scheme for teleporting an unknown, one-particle entangled state with a message passed in both directions between a sender (Alice) and a receiver (Bob) via a five-qubit cluster state. Our scheme used a one-way Hash function and XOR operations to blind the message. The security of the proposed schemes are guaranteed by the quantum one-time pad and quantum key distribution, which are different from the previous signature schemes in classical cryptography. In addition, compared with the existing quantum signature schemes, our schemes have some advantages. Firstly, unlike the quantum blind signature scheme, both schemes arranged eavesdropping checks to ensure security. Secondly, the first scheme used quantum teleportation to transmit two unknown qubit states, and were effective. Thirdly, owing to the maximum connectivity of cluster states and the persistence of entanglement, the second scheme was more secure. Fourthly, our scheme adopted BM and single-event measurements, which are easy to realize based on the existing technology and experimental conditions. Therefore, our scheme had a better security profile and could be applied to e-payment, e-commerce, e-voting, and other application scenarios.

However, the following problems still exist in quantum teleportation. (1) In quantum-teleportation experiments, the first step requires the preparation of the quantum channel (which is essentially the preparation of the quantum entangled state); the technology for preparing the multiparticle entangled state needs to be improved. (2) Moreover, when conducting quantum teleportation, the measurement of quantum states is a mandatory step, for which a high-precision measuring instrument is required. (3) Finally, when realizing the teleportation of quantum states, the particles need to pass through the quantum entanglement channel; during the process of transmission, various interference phenomena will occur. In other words, the teleportation process needs to be perfected to keep particles from being affected by external interference or physical factors in the process of transmission. The most prominent problem is the security problem after the combination of quantum teleportation and quantum signature. In the signature, we must prevent outside interferences, the infliction of harm on internal actors, and more importantly, the leakage of information. We believe that these problems can be overcome with further investigations.

## Data Availability

Data sharing not applicable to this article as no datasets were generated or analyzed during the current study.
